# Analysis of quantitative trait loci and candidate gene exploration associated with cold tolerance in rice (*Oryza sativa* L.) during the seedling stage

**DOI:** 10.3389/fpls.2024.1508333

**Published:** 2025-01-07

**Authors:** Sumin Jo, Seong-Gyu Jang, Sais-Beul Lee, Ji-Yoon Lee, Jun-Hyeon Cho, Ju-Won Kang, Yeongho Kwon, So-Myeong Lee, Dong-Soo Park, Soon-Wook Kwon, Jong-Hee Lee

**Affiliations:** ^1^ Department of Southern Area Crop Science, National Institute of Crop Science, Rural Development Administration (RDA), Miryang, Republic of Korea; ^2^ Planning and Coordination Division, National Institute of Crop Science, Rural Development Administration (RDA), Jeonju, Republic of Korea; ^3^ Department of Plant Bioscience, Pusan National University, Miryang, Republic of Korea

**Keywords:** cold tolerance, doubled haploid, quantitative trait loci, rice, seedling stage

## Abstract

Cold stress during the seedling stage significantly threatens rice (*Oryza sativa* L.) production, specifically in temperate climates. This study aimed to identify quantitative trait loci (QTLs) associated with cold tolerance at the seedling stage. QTL analysis was conducted on a doubled haploid (DH) population derived from a cross between the cold-sensitive *indica* cultivar 93-11 and the cold-tolerant *japonica* cultivar Milyang352. Phenotypic analysis was conducted over 2 years (2022–2023) under cold water treatment (13°C) at the Chuncheon Substation, South Korea. Cold tolerance scores were used to classify the DH populations and parental lines. In 2022, three QTLs were identified on chromosomes 3, 10, and 11; in 2023, a single QTL was identified on chromosome 10. The QTL *qCTS10^22/23^* on chromosome 10 was consistently observed across both years, explaining up to 16.06% and 40.55% of the phenotypic variance, respectively. Fine-mapping of *qCTS10^22/23^* narrowed the candidate region to a 300-kb interval containing 44 polymorphic single-nucleotide polymorphisms. Among the candidate genes, *Os10g0409400* was significantly expressed in the cold-tolerant *japonica* parent Milyang352 under cold stress, indicating its role in conferring cold tolerance. These findings offer valuable insights into the genetic mechanisms of cold tolerance and highlight *qCTS10^22/23^* as a potential target for marker-assisted selection in rice breeding programs to enhance cold tolerance.

## 1 Introduction

Cold-induced yellowing during the seedling stage significantly affects rice growth and yield, especially in temperate and high-altitude regions. *Indica* varieties, owing to their tropical origins, are particularly susceptible to low temperatures (Ahmad, 2022; Kim et al., 2021; Dasgupta et al., 2020; Zhang et al., 2014).

Cold stress at the seedling stage poses a significant threat to the growth and yield of cultivated rice. This stress can lead to substantial yield losses by impeding early growth, a critical phase that influences subsequent developmental stages. Cold stress has impacted rice yields across more than 25 countries, resulting in estimated annual losses of 3–5 billion kg and threatening global food security and economic stability (Cruz et al., 2013). In Northeast China, for example, the incidence of chilling damage to rice has increased more than 10-fold since the 1950s (Han et al., 2020). Similarly, in Korea, cold spells in April have frequently led to seedling rot and significant delays in growth, resulting in considerable seedling loss. Historical cold stress events in Korea, such as those in 1980 and 1993, led to yield reductions of approximately 26% and 9.2% of the national rice cultivation area compared to previous years, respectively (Jang et al., 2024; Jeong et al., 2020). Studies indicate that addressing cold stress during the seedling stage is essential for stabilizing rice yields, as early-stage cold tolerance enhances resilience and yield stability throughout the rice growth cycle, especially in regions where low temperatures frequently challenge crop productivity (Jia et al., 2019; Shi et al., 2022).

When exposed to temperatures below 15°C–20°C, rice seedlings experience chlorosis (yellowing of leaves), growth retardation, and a reduction in tiller number, resulting in substantial yield losses (Fujino et al., 2008). These physiological disturbances, often cold-induced yellowing, are crucial for rice production in temperate and high-altitude regions (Zhang et al., 2014). Therefore, addressing cold-induced stress during the seedling stage is essential for stabilizing rice yields in these vulnerable regions, particularly as climate variability increases the frequency of temperature fluctuations (Liu et al., 2022; Hossain et al., 2023).

Previous studies have highlighted the importance of understanding the genetic basis of cold tolerance in rice to develop varieties resilient to low temperatures (Andaya and Mackill, 2003). While *japonica* rice generally exhibits greater cold tolerance, many widely cultivated *indica* varieties are highly susceptible, underscoring the need for cold-tolerant breeding to ensure sustainable rice production under changing climatic conditions (Jiang et al., 2020).

Quantitative trait locus (QTL) mapping has proven to be a powerful tool for identifying genetic regions linked to cold tolerance in rice, particularly at the seedling stage. While various QTLs for seedling-stage cold tolerance have been reported, many are limited to specific environments and genetic backgrounds, complicating their application in breeding programs (Zhang et al., 2005; Lv et al., 2016; Zhou et al., 2024). For instance, QTLs associated with cold tolerance at the seedling stage have been mapped to chromosomes 9 and 12 (Zhang et al., 2014), with some showing significant effects across multiple environments (Zhang et al., 2014). Additionally, Jiang et al. (2020) identified QTLs related to cold tolerance on chromosomes 1, 3, 4, 6, 7, 9, 10, and 12, further illustrating the multi-chromosomal nature of this trait. Previous studies have identified QTLs associated with cold tolerance across most rice chromosomes, suggesting that this trait is controlled by multiple genetic loci distributed throughout the genome (Wang et al., 2011; Yang et al., 2020).

Cold tolerance in rice varies significantly between *japonica* and *indica* varieties (Dasgupta et al., 2020; Glaszmann et al., 1990; Mackill and Lei, 1997; Suh et al., 2012; Yang et al., 2023). Andaya and Tai (2006) identified a major QTL, *qCTS12*, on chromosome 12 using the cold-tolerant *japonica* variety M202 and the highly sensitive *indica* variety IR50. A fine-mapping analysis using 1,954 F_5_–F_10_ lines identified two candidate genes, *OsGSTZ1* and *OsGSTZ2*, associated with cold tolerance. Jiang et al. (2017) also studied cold stress using a combination of *japonica* and *indica* varieties, specifically 124 rice backcross recombinant inbred lines (BRILs) derived from a cross between the *indica* cultivar Changhui 891 and the *japonica* cultivar 02428. Based on a newly constructed high-density genetic map, they identified QTLs *qGRR1* and *qGRR8*, which affected the seed germination recovery rate after cold stress. They reported that these QTLs contained alleles with increasing effects. These findings contribute to a deeper understanding of the genetic basis for cold tolerance in rice and provide potential targets for further breeding research.

This study identified QTLs associated with cold tolerance at the seedling stage (CTS). QTL mapping was conducted using a doubled haploid (DH) population derived from a cross between the cold-sensitive *indica* cultivar 93-11 and cold-tolerant *japonica* line Milyang352. Phenotypic traits, such as growth parameters under cold stress, were analyzed to provide insights for marker-assisted selection (MAS) programs. Identifying stable QTLs for CTS across multiple environments can enhance cold tolerance in diverse rice backgrounds.

Understanding the genetic mechanisms underlying CTS has become a key research focus. MAS for cold tolerance offers a promising approach to mitigating the effects of low temperatures under changing climatic conditions. This study systematically identified CTS-associated QTLs in rice, using DH lines derived from a cross between a cold-sensitive *indica* cultivar and a cold-tolerant *japonica* line and assessing tolerance through cold water treatment (13°C for 10 days) during the seedling stage. The findings contribute valuable insights for MAS programs and highlight the need to further explore candidate genes within these QTL regions to deepen our understanding of the molecular mechanisms behind cold tolerance.

## 2 Materials and methods

### 2.1 Plant materials and mapping population for cold tolerance during the seedling stage

A population of 128 DH lines was derived from a cross between the typical *indica* rice cultivar 93-11 and the *japonica* cultivar Milyang352 (Lee et al., 2020). In the summer of 2016, F_1_ plants were generated, and their pollen was cultured on an N6Y2 medium to induce callus formation, from which regenerated plants were obtained. In the winter of 2016/2017, the regenerated A_0_ generation plants were cultivated in a greenhouse. In the summer of 2017, 128 A_1_ generation lines were selected to develop a DH population to assess the phenotypic data for cold tolerance during the seedling stage. Additionally, they were used to construct a molecular genetic map to identify QTLs that control survival under cold conditions during the seedling stage over 2 years (2022–2023).

### 2.2 Cold water treatment and cold-tolerance assessment

In 2022 and 2023, cold water treatment of rice seedlings was conducted at the Chuncheon Substation of the National Institute of Crop Science, RDA (Chuncheon, Korea). The rice seedlings were cultivated in 72-cell trays (measuring 3.8 cm × 3.8 cm × 4.5 cm) filled with paddy soil to mimic field conditions. The seedlings were grown until the three-leaf stage before initiating cold tolerance treatment. As control varieties, ‘Satbyeolbyeo’ (cold-sensitive) and ‘Seolagbyeo’ (cold-tolerant) were used to validate the cold tolerance assessment under these conditions. The rice seedlings were placed in a cold water storage facility in Chuncheon, which measured 50 m in length and 8 m in width and could supply cold water (13°C ± 1°C) through outlets spaced 70 cm apart with an 8-mm diameter. During the experiment, the average temperature in Chuncheon was approximately 20.0°C in 2022 and 20.2°C in 2023, with relative humidity (RH) ranging from 60% and 80% RH during the same periods, reflecting typical conditions during the experimental period. After 2 weeks of growth under normal greenhouse conditions, the trays were placed in the cold water treatment facility and rinsed with cold water for 10 days. Subsequently, the seedlings were recovered for 7 days in the greenhouse (Jiang et al., 2011; Kim et al., 2021) (**Supplementary Figure S1**). The phenotypes of the recovered seedlings were scored based on changes in leaf color, with scores ranging from 1 to 9, following the standard protocol provided by the International Rice Research Institute (IRRI), where 1 represents no change in leaf color, 5 indicates light yellow in half of the leaf, and 9 represents almost dead leaf (Donoso et al., 2015; IRRI, 2002).

### 2.3 DNA extraction and genotype analysis

Total genomic DNA was extracted from the fresh leaves of 4-week-old individual plants using the cetyltrimethylammonium bromide method, with certain modifications. DNA was quantified using a NanoDrop spectrophotometer (ND1000 spectrophotometer; Mettler Toledo, Greifensee, Switzerland). To construct the molecular map, Kompetitive allele-specific PCR (KASP) marker amplification and allelic discrimination were performed using the Nexar System (LGC Douglas Scientific, Alexandria, VA, USA) at the Seed Industry Promotion Center (Gimje, Korea) of the Foundation of Agri, Tech, Commercialization & Transfer in Korea. The KASP reaction mixture consisted of 0.8 μL of 2X master mix (LGC Genomics, London, UK), 0.02 μL of 72 KASP assay mix (LGC Genomics), and 5 ng genomic DNA template in a total volume of 1.6 μL in a 384-well array tape. KASP amplification was performed as described by Cheon et al. (2018). Fluidigm markers for single-nucleotide polymorphism (SNP) genotypes were determined using the BioMark™ HD system (Fluidigm, San Francisco, CA, USA) and 96.96 Dynamic Array IFC (96.96 IFC) chip, following the manufacturer’s protocol at the National Instrumentation Center for Environmental Management, Seoul National University (Pyeongchang, South Korea). Genotyping results were obtained using the Fluidigm SNP Genotyping Analysis software. A total of 240 SNP markers, including 106 KASP and 134 Fluidigm SNP markers, were used for genotyping the DH population. Sanger sequencing analysis was utilized to identify SNPs and insertion and deletion (InDel) regions that differ between the parental cultivars and explore candidate genes.

### 2.4 Construction of linkage map and QTL analysis

Linkage maps were constructed from genotype data using QTL IciMapping version 4.1 (Meng et al., 2015). Genetic distances were estimated using the Kosambi map function of the software (Kosambi, 1944). Additionally, over 2 years, QTL IciMapping was used for phenotypic data to analyze cold tolerance during the seedling stage. Inclusive composite interval mapping for additive QTL (ICIM-ADD) was used for analysis. The limit of detection (LOD) scores for ICIM-ADD were determined at an experiment-wise error rate of p > 0.05 by performing 1,000 permutations using QTL IciMapping.

### 2.5 Fine-mapping analysis of candidate region

The hybridization step followed the GeneTitan^®^ Multichannel Instrument User’s Manual using the KNU Axiom Oryza 580 K Genotyping Array. After ligation, the arrays were then stained and imaged using a GeneTitan MC Instrument (Kim et al., 2022). Image data were analyzed using the Affymetrix^®^ GeneChip^®^ Command Console^®^ software (Thermo Fisher Scientific, Waltham, MA, USA) to generate raw intensity values. Genotype calling was conducted using the Affymetrix Power Tools software. The BRLMM-P algorithm, which applies a Gaussian mixture model for one-dimensional clustering, was employed to ensure high accuracy in SNP genotype determination (BRLMM-P: a Genotype Calling Method for the SNP 5.0 Array). SNP markers with low call rates or poor clustering quality were excluded during quality control to ensure data reliability. For high-throughput SNP genotyping, the PolyHighResolution chip from the KNU Axiom Oryza 580 K Genotyping Array, which included 230,526 high-confidence SNP markers from 93-11 and Milyang352, was utilized. The identified QTL region was scanned to explore SNP markers exhibiting polymorphism between the parental lines. The fine-mapping analysis focused on these polymorphic SNP markers to narrow down the candidate region and improve the resolution of QTL localization. This approach allowed for the identification of specific SNP markers within the QTL region, which are critical for MAS and for identifying candidate genes underlying cold tolerance in rice.

### 2.6 RNA extraction and real-time PCR analysis

Total RNA was isolated from the leaves during the seedling stage using an RNeasy Plant Mini Kit (Qiagen, Hilden, Germany). DNase treatment and cDNA synthesis used a QuantiTect Reverse Transcription kit (Qiagen). The reactions were triplicated, with actin expression (*OsActin1* and *Os03g0718100*) as an internal baseline control. Samples were kept on ice until qRT-PCR analysis using QuantStudio 1 (Thermo Fisher Scientific, Boston, MA, USA). The run parameters followed a standard PCR protocol: reverse transcription at 50°C for 15 min, denaturation at 95°C for 2 min, followed by 50 amplification cycles with denaturation at 95°C for 15 s and annealing and extension at 60°C for 1 min, during which fluorescence was acquired. Data were analyzed using the QuantStudio software version 1.5.2 (Thermo Fisher Scientific, Boston). The cycle threshold was set to 0.05, and the baseline was automatically chosen. Two-tailed t-tests were performed using the GraphPad software to compare the samples. Statistical significance was set at p < 0.01.

### 2.7 Haplotype and genotype analyses for the KRICE_CORE population

For the haplotype analysis of the 137 KRICE_CORE population, genomic data were generated with an average coverage of approximately eightfold using the Illumina HiSeq 2500 Sequencing Systems Platform (Illumina Inc., San Diego, CA, USA). Raw reads were aligned to the rice reference genome (IRGSP 1.0) to generate genotype calls, ensuring high accuracy in the subsequent analyses. To create a reliable genotype dataset, haplotype analysis was performed using the parameters missing values <1%, minor allele frequency >5%, and heterozygosity ratio <5%, as implemented in the PLINK software. These thresholds were chosen to minimize data noise and enhance the accuracy of the haplotype associations. Phenotypic data for each variety were averaged to determine cold tolerance scores, which were subsequently used to identify haplotypes significantly associated with cold tolerance. The 137 KRICE_CORE population was also subjected to cold water treatment during the seedling stage, and the resulting cold tolerance scores were assessed following standardized protocols. A neighbor-joining tree (NJ-Tree) was constructed using the MEGA X software to visualize the genetic relationships among the varieties. The NJ-Tree was further processed in the Newick format and visualized using the Interactive Tree of Life (iTOL) tool for enhanced interpretability (Kumar et al., 2018; Letunic and Bork, 2011).

## 3 Results

### 3.1 Phenotypic analysis of rice seedling stage under cold water treatment

**Figure 1:**
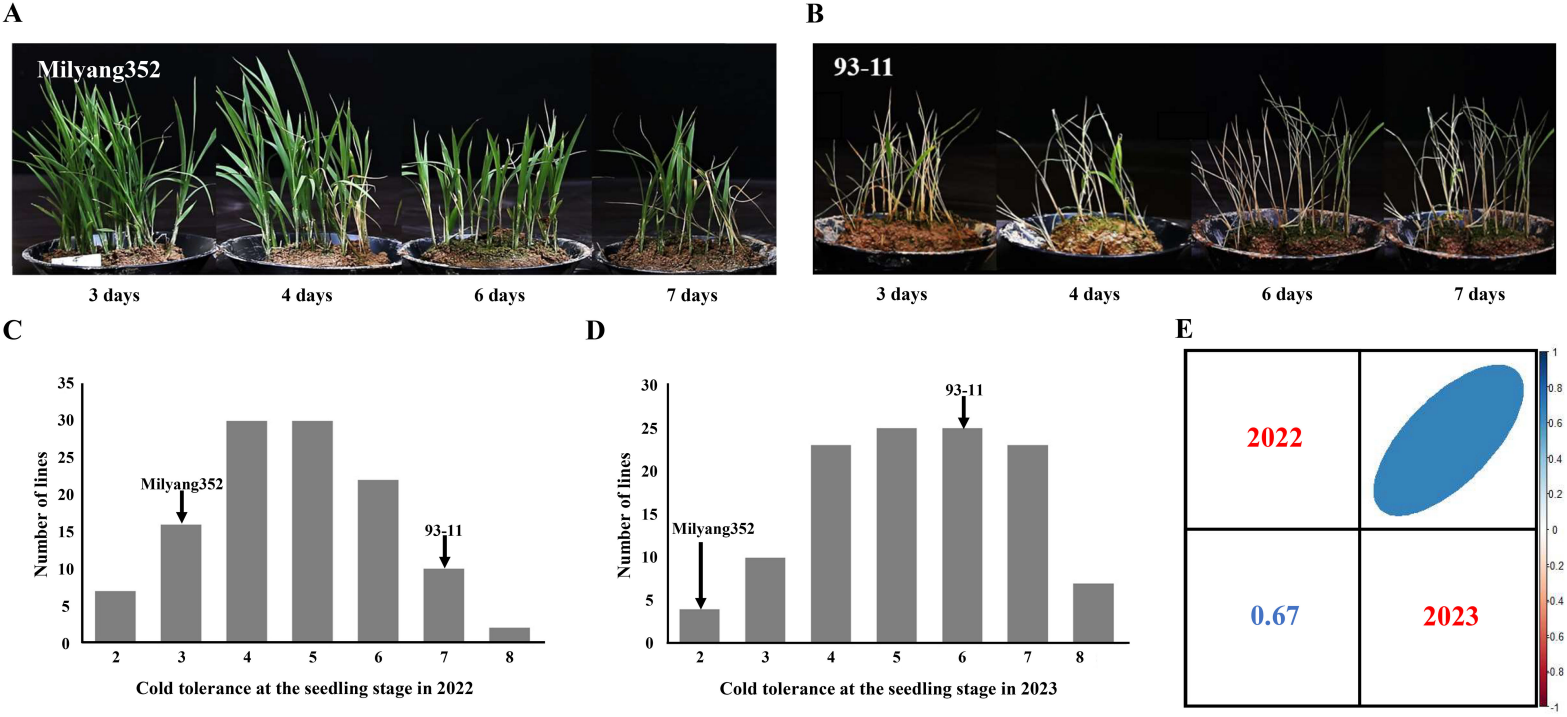
Phenotypic analysis of cold tolerance at the seedling stage in parental cultivars (93-11 and Milyang352) and 128 DH population. **(A)** Experimental results of Milyang352 under cold water treatment for 7 days. **(B)** Experimental results of 93-11 under cold water treatment during 7 days. **(C)** Frequency distributions of cold tolerance at the seedling stage in parental cultivars and DH population in 2022. **(D)** Frequency distributions of cold tolerance at the seedling stage in parental cultivars and DH population in 2023. **(E)** Pearson’s correlation of cold tolerance at the seedling stage during the 2-year period (2022–2023) using 128 DH population. DH, doubled haploid.

The cold tolerances of the rice varieties 93-11, Milyang352, and 128 DH lines were assessed during the seedling stage under cold water storage conditions over 2 years. Phenotypic analysis facilitated the classification of parent cultivars and DH populations into nine distinct types based on their cold tolerance scores. Across both years, the *indica* parent 93-11 consistently exhibited higher susceptibility to cold, with scores ranging from 6 to 7, whereas the *japonica* parent Milyang352 exhibited strong cold tolerance, with scores ranging from 2 to 3 (**Figures 1A–D**). In 2022, 23 DH lines were identified as cold-tolerant during the seedling stage (scores of 1–3), whereas 34 DH lines were classified as cold-susceptible (scores of 7–9) (**Figure 1C**). In 2023, a similar pattern was observed, with 14 DH lines exhibiting cold tolerance (scores 1–3) and 30 DH lines exhibiting susceptibility (scores 7–9) (**Figure 1D**). **Figure 1E** illustrates the variability among the DH populations across the 2 years. Despite this variability, a significant positive correlation was observed between the cold tolerance scores in 2022 and 2023 (r = 0.67, p-value < 0.01), indicating a consistent response to cold stress in the DH population across different years.

### 3.2 QTL analysis for cold tolerance during the seedling stage

To identify the genetic loci associated with cold tolerance during the seedling stage in the DH population, a linkage map was constructed, and QTL analysis was performed using ICIM analysis. Among the KASP and Fluidigm markers tested, 229 SNPs exhibited polymorphic patterns between parents. These markers were used to construct linkage maps from genotype data using QTL IciMapping version 4.1 (**Supplementary Figure S2**). Additionally, QTL IciMapping was used for QTL analysis using the phenotypic data, specifically through ICIM-ADD. The LOD threshold was obtained based on a permutation test (1,000 permutations, p = 0.05) for each trait.

**Figure 2:**
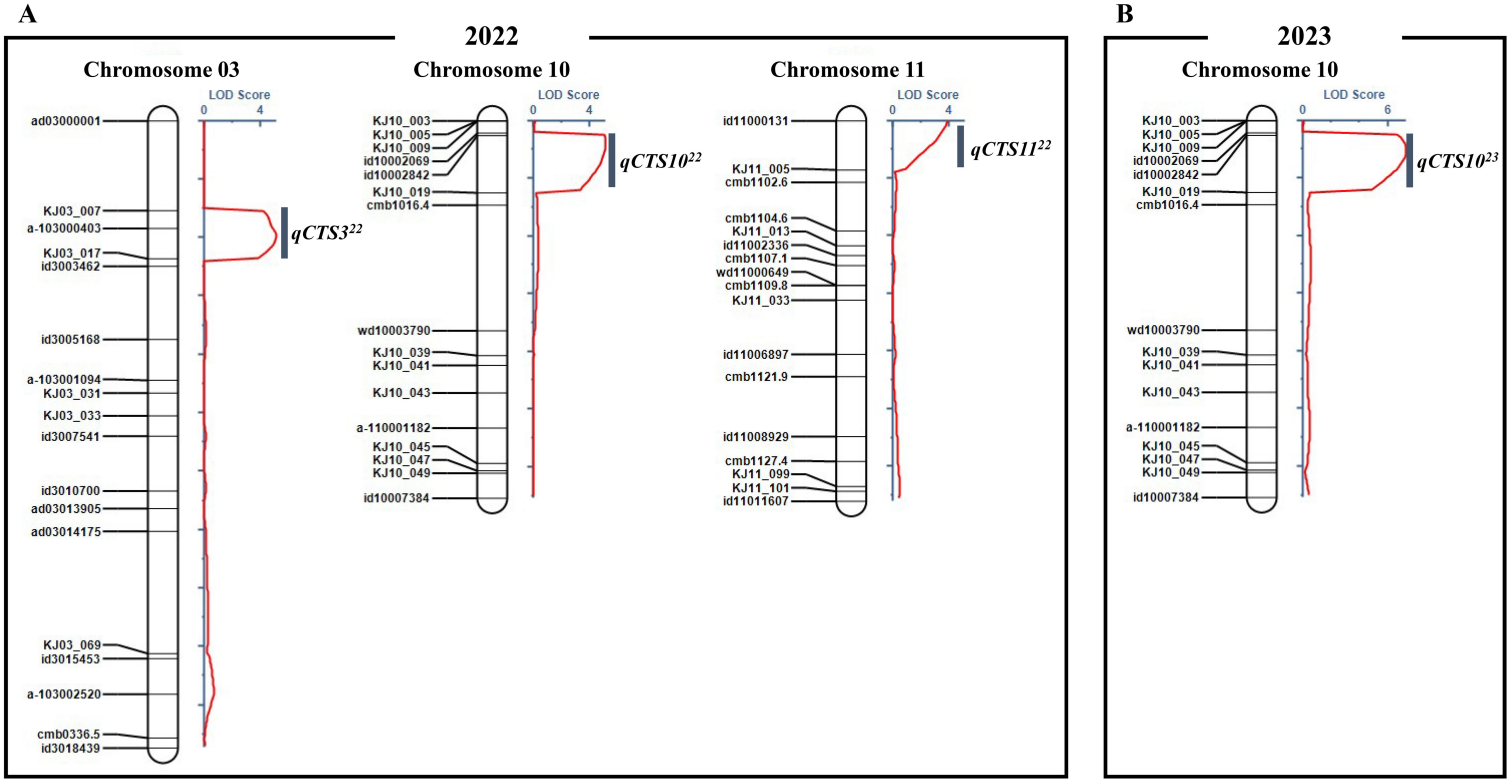
Detected QTL regions associated with cold tolerance at the seedling stage using DH population. **(A)** LOD score of DNA markers associated with cold tolerance at the seedling stage in 2022 and position of *qCTS3^22^*, *qCTS10^22^*, and *qCTS11^22^* on chromosomes 3, 10, and 11. **(B)** LOD score of DNA markers associated with cold tolerance at the seedling stage in 2023 and position of *qCTS10^23^* on chromosome 10. QTL, quantitative trait locus; DH, doubled haploid; LOD, limit of detection.

In the QTL analysis, three QTLs for cold tolerance during the seedling stage were detected on chromosomes 3, 10, and 11 in 2022, and one QTL was detected on chromosome 10 in 2023. In 2022, the QTL *qCTS3^22^* (LOD score = 5.09) was mapped on chromosome 3 (from 3,524 to 5,676 kb), accounting for 16.90% of the total phenotypic variance. On chromosome 10, *qCTS10^22^* (LOD score = 5.11) was mapped from 10,730 to 15,128 kb, accounting for 16.06% of the total phenotypic variance. On chromosome 11, *qCTS11^22^* (LOD score = 3.88) was mapped from 680 to 2,455 kb, accounting for 11.38% of the total phenotypic variance. In 2023, *qCTS10^23^* (LOD score = 16.70) was located on chromosome 10, at a position similar to that of *qCTS10^22^* from 2022, accounting for 40.55% of the phenotypic variance. The allele *qCTS11^22^* was confirmed to have been derived from 93-11, and the remaining QTLs were confirmed to have been derived from Milyang352 (**Figure 2**, **Table 1**). Based on the QTL analysis conducted over 2 years, the analysis focused on QTLs detected at the same loci (*qCTS10^22^* and *qCTS10^23^*).

**Table 1:** QTLs associated with cold tolerance during the seedling stage and identified using the DH population in 2022 and 2023.

QTLs	Year	Chromosome	Marker interval	LOD	PVE (%)	Add
*qCTS3^22^*	2022	3	a-103000403/KJ03_017	5.09	16.90	−0.33
*qCTS10^22^*	2022	10	id10002842/KJ10_019	5.11	16.06	−0.33
*qCTS11^22^*	2022	11	id11000131/KJ11_005	3.88	11.38	0.27
*qCTS10^23^*	2023	10	id10002842/KJ10_019	16.70	40.55	−0.80

QTLs, quantitative trait loci; DH, doubled haploid; LOD, limit of detection; PVE, phenotypic variation explained.

### 3.3 Fine-mapping analysis of *qCTS10^22^* and *qCTS10^23^*

**Figure 3:**
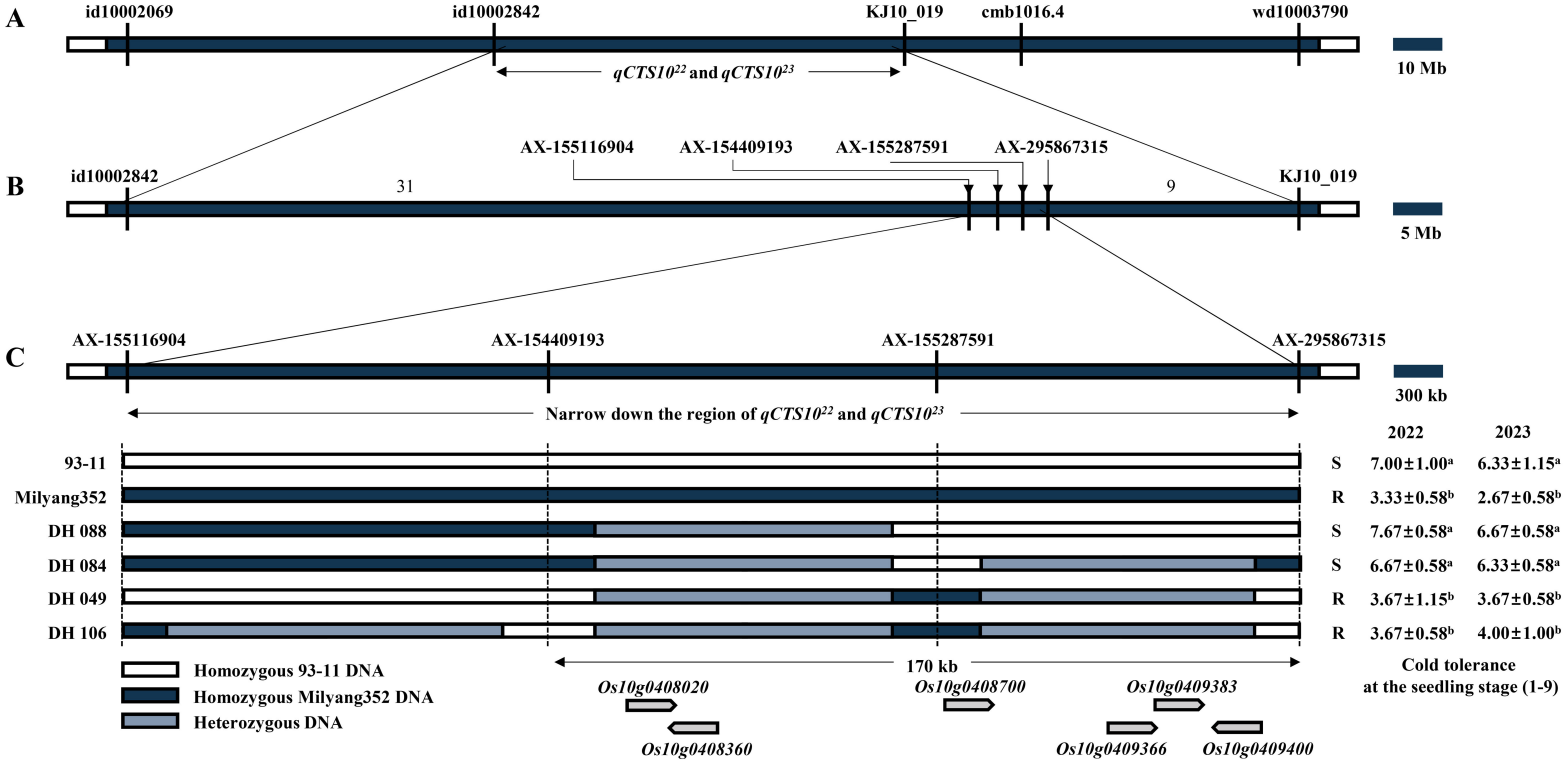
Fine-mapping of the *qCTS10^22^* and *qCTS10^23^*. **(A)** Physical map of the *qCTS10^22^* and *qCTS10^23^* locus using DH population. **(B)** High-resolution mapping of the *qCTS10^22^* and *qCTS10^23^* locus using DH population. **(C)** Recombination events and their effects on tolerance phenotypes in the *qCTS10^22^* and *qCTS10^23^* genotypes. Values represent means ± standard deviation. Letters a and b represent significant differences at p < 0.01 (Duncan’s test). DH, doubled haploid.

**Figure 4:**
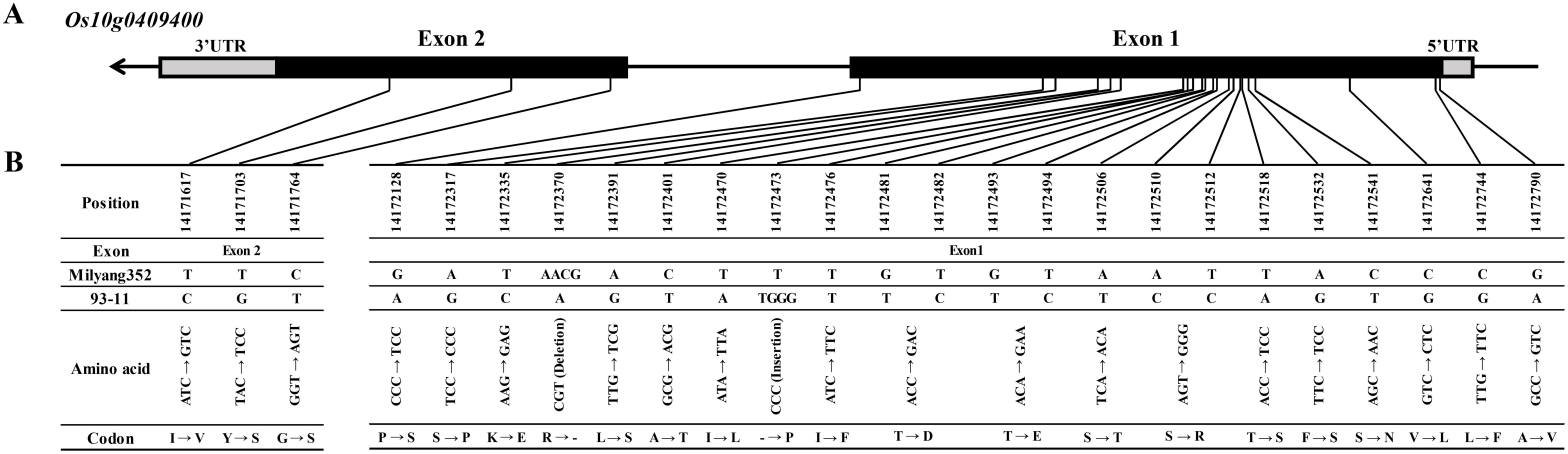
Gene structure and cDNA sequence comparison of *Os10g0409400*. **(A)** Schematic representation of the gene structure, SNP, and InDel positions in *Os10g0409400*. **(B)** *Os10g0409400* structure and cDNA sequence comparison. The black and gray block lines indicate the exons and untranslated regions, respectively. SNP, single-nucleotide polymorphism; InDel, insertion and deletion.

To narrow down the candidate region associated with cold tolerance during the seedling stage, a fine-mapping analysis of the allele *qCTS10^22/23^* on chromosome 10 was conducted using a 580 K SNP array (Kim et al., 2022). Polymorphic SNPs within the 580 K SNP array were identified by comparing the sequences of the parent cultivars (93-11 and Milyang352) between markers id10002842 and KJ10_019 on chromosome 10. This analysis confirmed the presence of 44 SNPs within this allele. These 44 SNPs were subsequently used for QTL dissection, facilitating further refinement of the candidate region using phenotypic data collected over 2 years. Therefore, the interval narrowed significantly from 5 Mb to 300 kb. The fine-mapping focused on four primary SNPs between AX-155116904 and AX-295867315 that are crucial for reducing the candidate region. The refined target region now encompasses a 170-kb interval with six predicted genes (*Os10g0408020*, *Os10g0408360*, *Os10g0408700*, *Os10g0409366*, *Os10g0409383*, and *Os10g0409400*) according to The Rice Annotation Project Database (https://rapdb.dna.affrc.go.jp/) (**Figure 3**). Among the six candidate genes of *qCTS10^22^* and *qCTS10^23^*, three candidate genes (*Os10g0408020*, *Os10g0408360*, and *Os10g0408700*) were monomorphic between 93-11 and Milyang352. The remaining three genes (*Os10g0409366*, *Os10g0409383*, and *Os10g0409400*) were sequenced to identify their respective regions. The *Os10g0409366* gene showed six SNPs, *Os10g0409383* had one SNP, and *Os10g0409400* exhibited 23 SNPs and two InDel regions between the parents (**Table 2**, **Figure 4**; **Supplementary Figure S3**).

**Table 2:** Candidate genes with cold tolerance for the *qCTS10^22/23^* at the seedling stage.

Gene name	Length of CDS (bp)	Putative function	Reported gene	Sequence variation
*Os10g0408020*	303	Predicted gene	–	–
*Os10g0408360*	261	Predicted gene	–	–
*Os10g0408700*	237	Similar to GAMYB-binding protein	–	–
*Os10g0409366*	1152	Predicted gene	–	Six SNPs
*Os10g0409383*	495	Predicted gene	–	One SNP
*Os10g0409400*	1035	Beta subunit of polygalacturonase 1; abiotic stress response	*OsRD22*, *OsBURP16*	23 SNPs and two InDel regions

CDS, coding sequence; SNPs, single-nucleotide polymorphisms; InDel, insertion and deletion.

### 3.4 Expression of candidate gene associated with cold tolerance during the seedling stage

To identify genes associated with cold tolerance at the seedling stage, real-time PCR was used to assess gene expression in leaves after 0, 12, and 24 h of cold treatment at the seedling stage. The expression levels of the candidate genes *Os10g0409366*, *Os10g0409383*, and *Os10g0409400*, located in the fine-mapped region associated with cold tolerance, were assessed using a primer set designed based on their coding sequences. The results demonstrated that *Os10g0409400* was significantly upregulated in the Milyang352 cultivar compared to that in the 93-11 cultivar, specifically after 12 h and 24 h of cold treatment (p < 0.01). At 12 h, Milyang352 exhibited expression levels approximately 12 times higher than those of 93-11, indicating a robust transcriptional response to cold stress. This increased expression persisted at 24 h, further supporting the association of *Os10g0409400* with cold tolerance in Milyang352.

**Figure 5:**
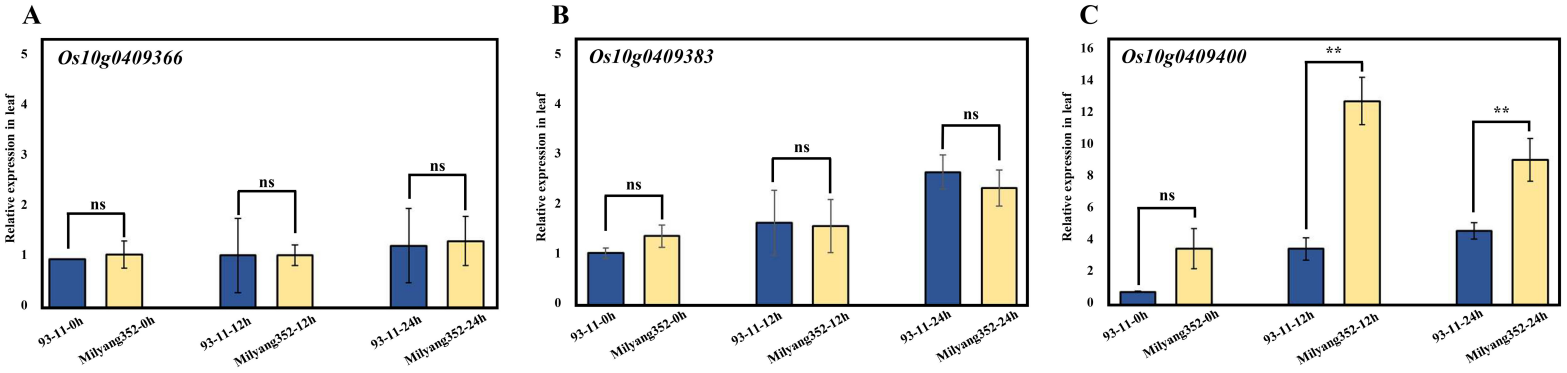
Expression analysis of candidate gene. **(A)** *Os10g0409366* expression in 93-11 and Milyang352 at seedling stage. **(B)** *Os10g0409383* expression in 93-11 and Milyang352 at seedling stage. **(C)** *Os10g0409400* expression in 93-11 and Milyang352 at seedling stage. Values were normalized against the level of *OsAct1*. Error bars represent standard deviations (n = 3), **p < 0.01. SNP, single-nucleotide polymorphism; InDel, insertion and deletion.

In contrast, *Os10g0409366* and *Os10g0409383* showed no significant differences in expression levels between the two cultivars (**Figure 5**). Additionally, 93-11 exhibited relatively low and stable expression levels of *Os10g0409400* across all time points, indicating a weaker response to cold stress. These findings indicate that *Os10g0409400* plays a crucial role in the cold tolerance observed in Milyang352 during the seedling stage. The significant differential expression between the two cultivars, specifically under prolonged cold stress, highlights the potential of *Os10g0409400* as a primary genetic determinant for enhancing cold tolerance in rice.

### 3.5 Haplotype analysis of 137 KRICE_CORE population

**Figure 6:**
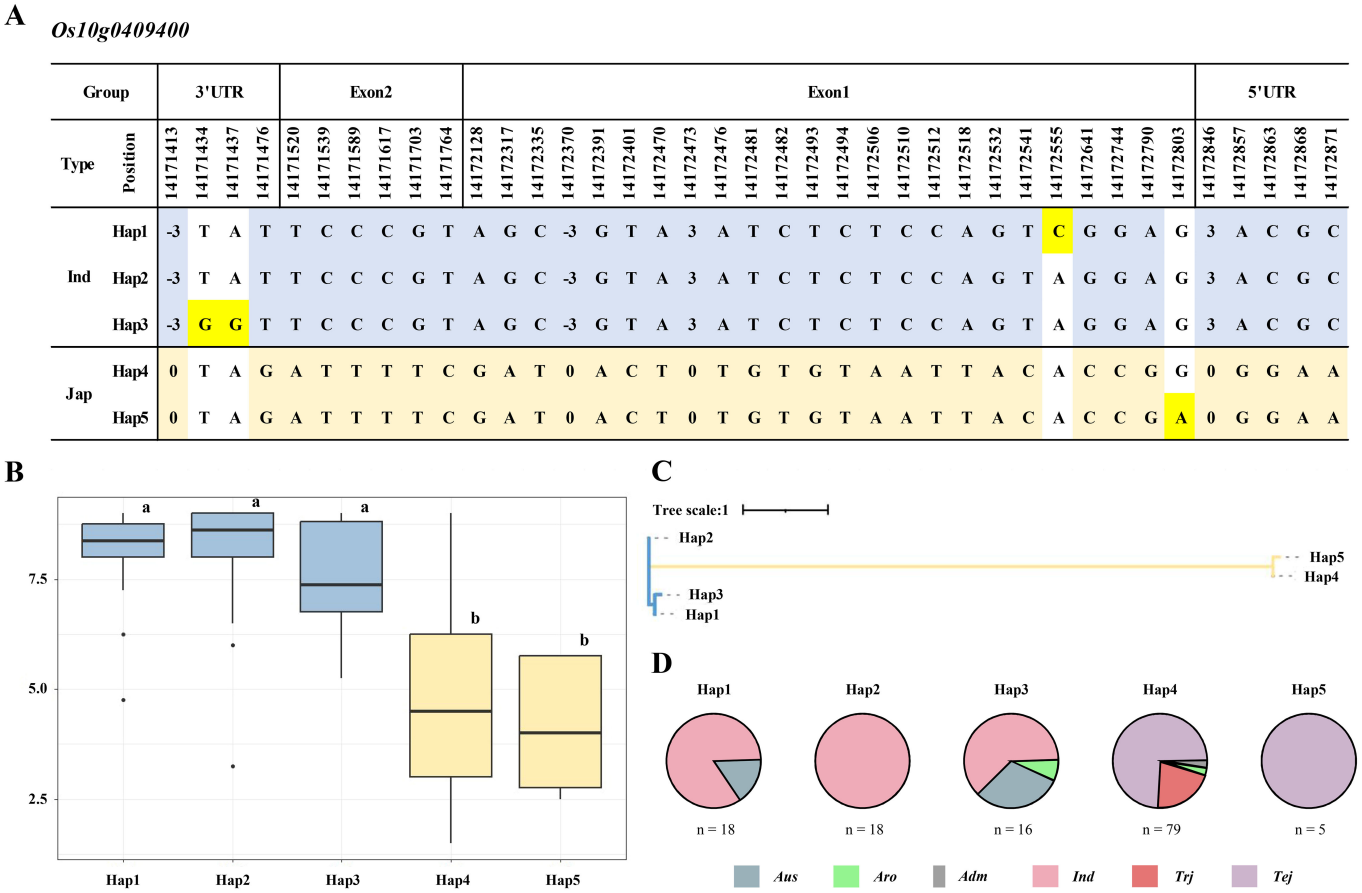
Haplotype analysis of *Os10g0409400*. **(A)** Haplotype analysis of *Os10g0409400* in 137 KRICE_CORE population. The blue- and mustard-shaded SNPs or InDels represent polymorphisms in the *indica* and *japonica* types. The yellow highlighted bases are observed only in Hap1, Hap3, and Hap5. **(B)** Boxplot displaying the phenotypic distribution of cold-tolerance scores among various haplotypes (Hap1–Hap5). Letters a and b represent significant differences at **p < 0.01 (Duncan’s test). **(C)** Neighbor-joining tree representing the evolutionary relationship between the identified haplotypes (Hap1–Hap5) based on sequence variations in the *Os10g0409400* gene. The tree scale indicates the genetic distance. **(D)** Pie charts illustrating the distribution of identified haplotypes (Hap1–Hap5) across various rice subspecies. Aro, *aromatic*; Adm, *admixture*; Ind, *indica*; Trj, *tropical japonica*; and Tej, *temperate japonica*. SNPs, single-nucleotide polymorphisms; InDels, insertions and deletions.

To explore the genetic diversity and potential associations with cold tolerance within the KRICE_CORE rice population, we performed a haplotype analysis of the *Os10g0409400* gene. We selected this gene because of its location within a QTL region associated with cold tolerance during the seedling stage. We identified five distinct haplotypes of *Os10g0409400* within the 137 KRICE_CORE population based on 35 SNPs and four InDels, with specific variations highlighted in blue and mustard to represent *japonica* and *indica* types, respectively (**Figure 6A**). The haplotype analysis revealed that among the five identified haplotypes, Hap1, Hap2, and Hap3 were predominantly associated with the *indica* type, whereas Hap4 and Hap5 were associated with the *japonica* type. Specifically, the *indica* parent 93-11 obtained from the DH population was classified under Hap2, and the *japonica* parent Milyang352 was classified under Hap4. The cold tolerance scores for Hap1, Hap2, and Hap3 were 7.90, 8.01, and 7.40, respectively, indicating susceptibility to cold stress during the seedling stage. In contrast, Hap4 and Hap5 exhibited lower scores, indicating a higher tolerance to cold stress (**Figure 6B**). Neighbor-joining tree analysis further categorized the 137 rice varieties into two distinct subgroups. The first subgroup included Hap1, Hap2, and Hap3, representing *indica* types, whereas the second subgroup included Hap4 and Hap5, representing *japonica* types (**Figure 6C**). The haplotype analysis demonstrated that Hap1 and Hap2 included 18 varieties each, with Hap1 comprising 3 and 15 Aus and *indica* types, respectively. Hap2 had entirely *indica* types, whereas Hap3 included 16 varieties with five Aus types, one aromatic, and 10 *indica* types. Hap4, the most diverse haplotype, also included 79 varieties with two aromatic types, one admixture type, 57 temperate *japonica* types, and 19 tropical *japonica* types. Hap5 contained five varieties, all temperate *japonica* types (**Figure 6D**).

## 4 Discussion

This study identified several QTLs associated with cold tolerance during the seedling stage in a DH population derived from a cross between *indica* variety 93-11 and *japonica* variety Milyang352. Cold tolerance is an important trait for rice cultivation in temperate regions, where low temperatures during the early growth stages can result in significant yield losses. Our findings contribute to understanding the genetic mechanisms underlying cold tolerance in rice and offer a basis for future breeding programs aimed at enhancing cold resistance.

The consistent detection of *qCTS10^22/23^* on chromosome 10 in both 2022 and 2023 highlights the stability and significance of this locus in conferring cold tolerance. It should be noted that the 13°C cold water treatment used in this study provides a stable and controlled environment for assessing cold tolerance; however, it may not fully represent the variable temperature conditions that rice seedlings encounter in natural field environments. This limitation highlights the need for future studies to include fluctuating temperature treatments to better mimic real-world conditions, which could enhance the applicability of these findings. Controlled low-temperature treatments, like the one used here, are effective in distinguishing genotypic differences in cold tolerance under laboratory conditions (Fujino et al., 2008), serving as a foundational step for further investigation. Additionally, the identification of *qCTS10^22/23^* across multiple years suggests its potential utility in breeding programs to enhance rice’s cold tolerance. Breeders could select cold-tolerant genotypes early by incorporating markers linked to this QTL in MAS, thus streamlining the breeding process.

Identifying *qCTS3^22^* and *qCTS11^22^* on chromosomes 3 and 11 further supported the polygenic nature of cold tolerance in rice, respectively. Compared to the findings of previous studies, our findings provide a more stable identification of *qCTS10^22/23^* across different years and environments, highlighting its potential as a consistent target for breeding. For instance, earlier studies identified QTLs related to cold tolerance, but many were limited to specific genetic backgrounds or environmental conditions, restricting their broader applicability (Xu et al., 2008; Jiang et al., 2017). In contrast, our consistent detection of *qCTS10^22/23^* in both 2022 and 2023 suggests that this locus may offer greater stability across varying conditions, which is advantageous for practical breeding applications. Furthermore, identifying *Os10g0409400* within this QTL region adds a functional layer, as this gene’s significant upregulation under cold stress directly enhances cold tolerance, potentially advancing the understanding of molecular mechanisms in rice. Although these QTLs accounted for lower proportions of the phenotypic variance than *qCTS10^22^*, their detection in 2022 indicates that multiple genetic loci contribute to the complex trait of cold tolerance. *qCTS3^22^* corresponds to the previously reported *qCTS3-9* region, which was identified through a genome-wide association study (GWAS) using 295 rice cultivars from the rice diversity panel 1 (Wang et al., 2016). Similarly, Li et al. (2021) identified three QTLs (*qSR3-1*, *qSR3-2*, and *qSR3-3*) associated with cold tolerance at the bud burst stage, which is also located within the *qCTS3^22^* allele, through GWAS analysis of a natural population of 211 rice accessions. Xu et al. (2008) identified eight QTLs across five chromosomes using 1,557 BC_5_F_2_ near-isogenic lines, and *qCTS10^22/23^* was found in the same region as *qCTB-10-1* on chromosome 10. Furthermore, Liu et al. (2020) detected *qESCT-10* at the early seedling stage, using 172 recombinant inbred lines (RILs), which overlaps with the *qCTS10^22/23^* locus, further supporting the presence of cold tolerance loci on this chromosome. Lastly, *qCTS11^22^* overlapped with the *Q14d-11* and *qSCT-11* regions, identified using 120 DH lines and 269 RILs, respectively (Ji et al., 2010; Zhang et al., 2005) (**Table 3**). Further studies must confirm these QTLs in different environments and assess their interactions with other loci.

**Table 3:** QTLs associated with cold tolerance at the seedling stage reported in previous studies.

QTLs	QTL accession	Population	Related trait	Reference
*qCTS3^22^*	*qCTS3-9*	295 rice diversity panel 1	Cold tolerance at the seedling stage	Wang et al., 2016
	*qSR3-1~3*	211 natural population	Cold tolerance at the bud burst stage	Li et al., 2021
*qCTS10^22/23^*	*qCTB-10-1*	1,557 BC_5_F_2_	Cold tolerance at the boosting stage	Xu et al., 2008
	*qESCT-10*	172 RILs	Cold tolerance at the early seedling stage	Liu et al., 2020
*qCTS11^22^*	*Q14d-11* *qSCT-11*	120 DH lines 269 RILs	Cold tolerance at the seedling stage	Ji et al., 2010 Zhang et al., 2005

QTLs, quantitative trait loci; RILs, recombinant inbred lines.

The fine-mapping of *qCTS10^22/23^* narrowed the candidate region to a 300-kb interval containing 44 polymorphic SNPs. This precision is crucial for identifying candidate genes directly involved in cold tolerance. Our gene expression analysis identified *Os10g0409400* as a strong candidate because its expression was significantly upregulated in cold-tolerant Milyang352 during cold stress.

*Os10g0409400*, also known as *OsRD22* and *OsBULP16*, is a gene whose transcription is reported to be induced by cold, salinity, drought stresses, and abscisic acid treatment. *Os10g0409400* is reported to have high expression levels during the seedling stage, causing pectin degradation and affecting cell wall integrity and transpiration rate, reducing tolerance to abiotic stresses (Liu et al., 2014). Haplotype analysis showed a distinction between the *indica* and *japonica* types, suggesting that this gene may significantly influence the degree of cold tolerance during the seedling stage between the two types. The role of this gene in cold tolerance requires further assessment, including functional validation through gene knockout or overexpression experiments. While this study primarily focused on cold tolerance, it is worth considering how the identified QTLs, particularly *qCTS10^22/23^*, may interact with other abiotic stress factors, such as drought or salinity. Previous studies have shown that some genes involved in cold tolerance also respond to other stressors due to shared regulatory pathways (Liu et al., 2014). Future research could explore the response of *qCTS10^22/23^* and its associated genes under multiple stress conditions, providing a more comprehensive understanding of their potential utility in breeding programs to improve overall stress resilience in rice. The candidate gene *Os10g0409400* likely plays a crucial role in the cold tolerance mechanism through its function in cell wall stability and water regulation under stress conditions. *Os10g0409400* belongs to the BURP domain-containing protein family and is associated with pectin degradation in the cell wall, affecting cell wall integrity (Ding et al., 2009). This function is particularly important under cold stress, as maintaining cellular integrity and reducing water loss are critical for survival. Additionally, the significant upregulation of *Os10g0409400* during cold stress suggests that it may contribute to a rapid adaptive response by stabilizing cell walls and modulating transpiration rates. However, further studies, such as gene knockout or overexpression analyses, must confirm its precise role in the cold tolerance pathway.

The observed higher susceptibility of 93-11 than Milyang352 to cold stress is consistent with the findings of previous studies, which indicate that *japonica* varieties generally have greater cold tolerance than *indica* varieties (Andaya and Mackill, 2003; Lv et al., 2016). This difference in cold tolerance between the two subspecies provides a valuable opportunity for breeding, as our study identified QTLs derived from both parents. Combining favorable alleles from both *indica* and *japonica* subspecies could potentially lead to the development of rice varieties with enhanced cold tolerance, which is particularly beneficial for regions affected by low-temperature stress. Despite the insights provided by this study, some limitations should be addressed in future research. First, while effective in a controlled setting, the 13°C cold water treatment used here may not fully capture the natural temperature fluctuations rice seedlings experience in field conditions. Future studies could implement variable temperature treatments to better mimic field environments, potentially enhancing the applicability of our findings. Additionally, while this study identified key QTLs and candidate genes associated with cold tolerance, further functional validation of these genes, such as gene knockout or overexpression studies, is necessary to confirm their roles in cold tolerance. Lastly, assessing the interactions between these QTLs and other abiotic stress factors, such as drought or salinity, could provide a more comprehensive understanding of their utility in breeding programs to improve overall stress resilience in rice.

In conclusion, our study identified and confirmed primary QTLs associated with cold tolerance during the seedling stage, specifically *qCTS10^22/23^*, which exhibits strong potential for use in rice breeding programs. Identifying candidate genes, particularly *Os10g0409400*, presents valuable opportunities for implementing MAS. Using molecular markers linked to these genes, breeders can efficiently screen for cold-tolerant genotypes at early stages, thus accelerating the breeding process. *Os10g0409400*, significantly upregulated under cold stress, could be a reliable marker for selecting resilient lines before field testing. Incorporating these genes into MAS would facilitate the development of rice varieties adapted to temperate climates, ensuring stable yields under fluctuating environmental conditions by reducing the need for extensive phenotypic screening. Identifying candidate genes within this region paves the way for further research into the molecular mechanisms of cold tolerance in rice. These findings offer valuable genetic resources that can be used to develop rice varieties capable of thriving in colder climates, ensuring stable yields under fluctuating environmental conditions.

## Data Availability

The original contributions presented in the study are included in the article/**Supplementary Material**, further inquiries can be directed to the corresponding authors.
