# Supplementary figures and images for: Analysis of quantitative trait loci and candidate gene exploration associated with cold tolerance in rice (Oryza sativa L.) during the seedling stage
Source: Front Plant Sci. 2025 Jan 7;15:1508333. doi: 10.3389/fpls.2024.1508333 (PMC11747135; doi:10.3389/fpls.2024.1508333)

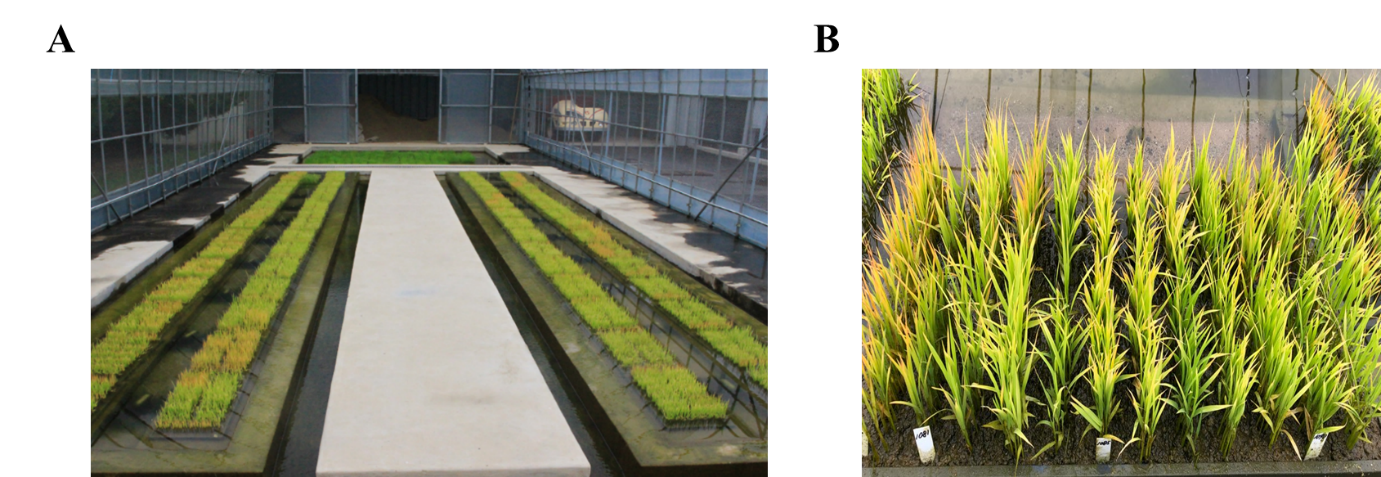

Supplement: Supplementary file 1 [file Table1.docx]

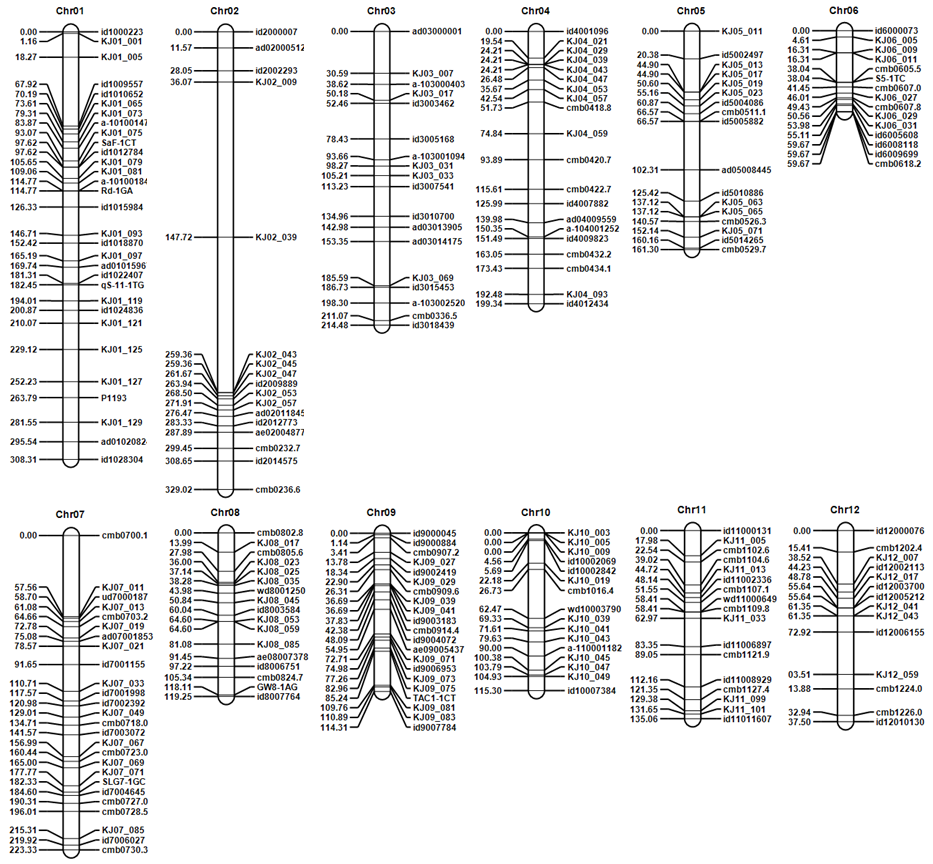

Supplement: Supplementary file 2 [file Table2.docx]

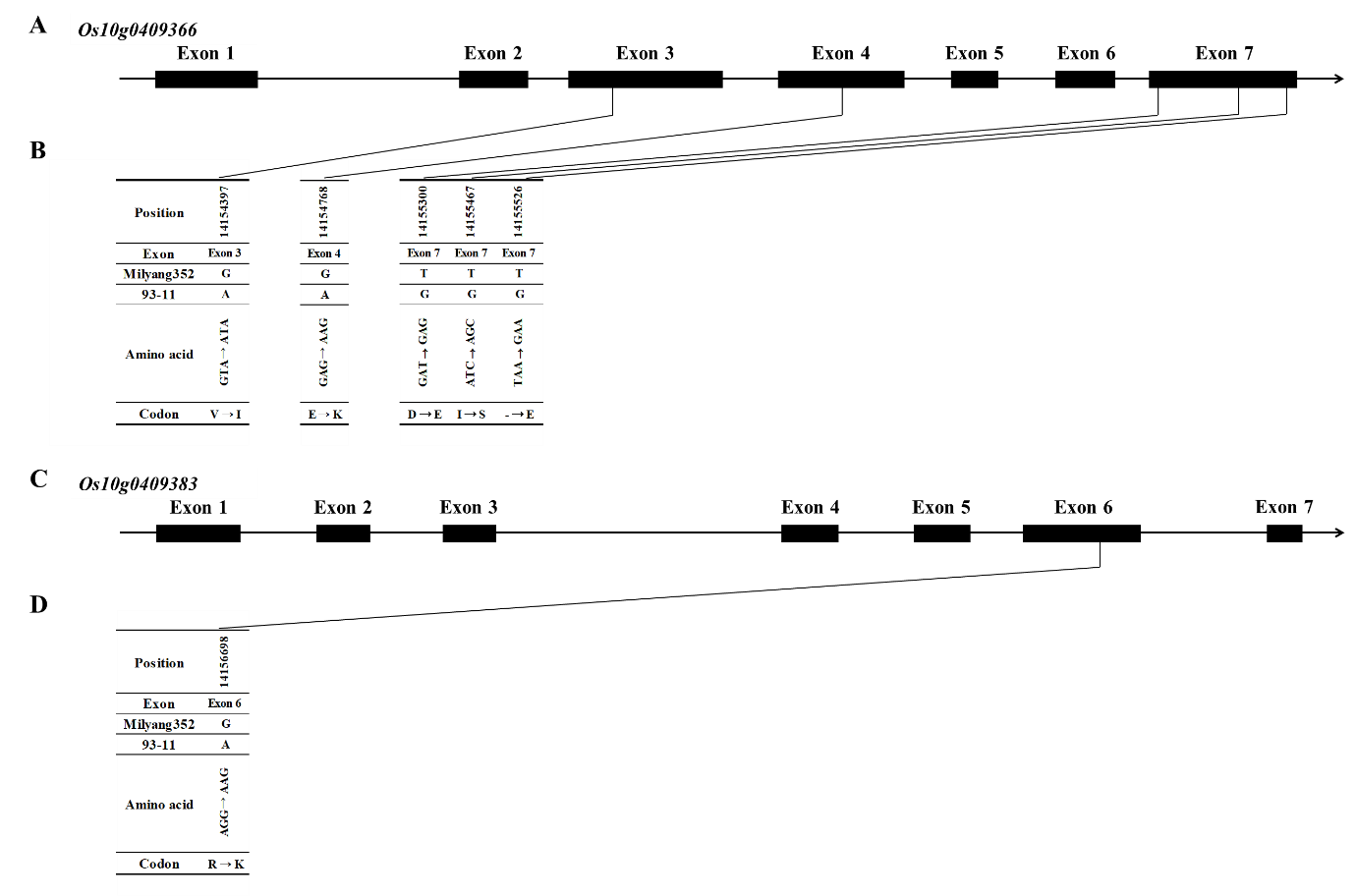

Supplement: Supplementary file 3 [file Table3.docx]
